# Theory of the Flower Micelle Formation of Amphiphilic Random and Periodic Copolymers in Solution

**DOI:** 10.3390/polym10010073

**Published:** 2018-01-14

**Authors:** Takahiro Sato

**Affiliations:** Department of Macromolecular Science, Osaka University, 1-1 Machikaneyama-cho, Toyonaka, Osaka 560-0043, Japan; tsato@chem.sci.osaka-u.ac.jp; Tel.: +81-6-6850-5461

**Keywords:** amphiphilic polymers, random copolymers, alternating copolymers, flower micelles, flower necklaces, vesicle

## Abstract

The mixing Gibbs energy Δ*g*_m_ for the flower-micelle phase of amphiphilic random and periodic (including alternating) copolymers was formulated on the basis of the lattice model. The formulated Δ*g*_m_ predicts (1) the inverse proportionality of the aggregation number to the degree of polymerization of the copolymer, (2) the increase of the critical micelle concentration with decreasing the hydrophobe content, and (3) the crossover from the micellization to the liquid–liquid phase separation as the hydrophobe content increases. The transition from the uni-core flower micelle to the multi-core flower necklace as the degree of polymerization increases was also implicitly indicated by the theory. These theoretical results were compared with experimental results for amphiphilic random and alternating copolymers reported so far.

## 1. Introduction

Borisov and Halperin [1,2,3,4,5] proposed theoretical models of flower micelles, flower necklaces, and bouquets of polymer micelles formed by amphiphilic periodic copolymers composed of hydrophilic and hydrophobic monomer units in aqueous solutions. They assumed that the main chain of the periodic copolymer is perfectly flexible, and all hydrophobes in the copolymer chain are included in the hydrophobic core(s) of the micelle.

Afterward, experimental studies on amphiphilic random and periodic (including alternating) copolymers bearing hydrophobic side chains demonstrated the formation of flower micelles and flower necklaces in aqueous solutions [6,7,8,9,10,11]. However, experimental results indicated that not all hydrophobic side chains on the copolymer chain are included in the hydrophobic core(s) of the micelle, being different from the Borisov–Halperin model, and that the loop-chain size is determined by the main-chain stiffness rather than the content and sequence of the hydrophobic side chain on the copolymer chain. Thus, we need a new theory to discuss the micellization behavior of such flower micelles and flower necklaces.

The present paper proposes a lattice-model theory for dilute aqueous solutions of amphiphilic random and periodic copolymers bearing hydrophobic linear side chains, which can be regarded as graft copolymer chains bearing hydrophobic graft (side) chains to demonstrate the formation of the flower micelle. Recently, Sato and Takahashi [12] presented a similar lattice-model theory for amphiphilic block copolymer solutions to discuss the competition between micellization and liquid–liquid phase separation in the solutions. The present theory is the random and periodic copolymer version of this theory.

## 2. Theory

**Figure 1:**
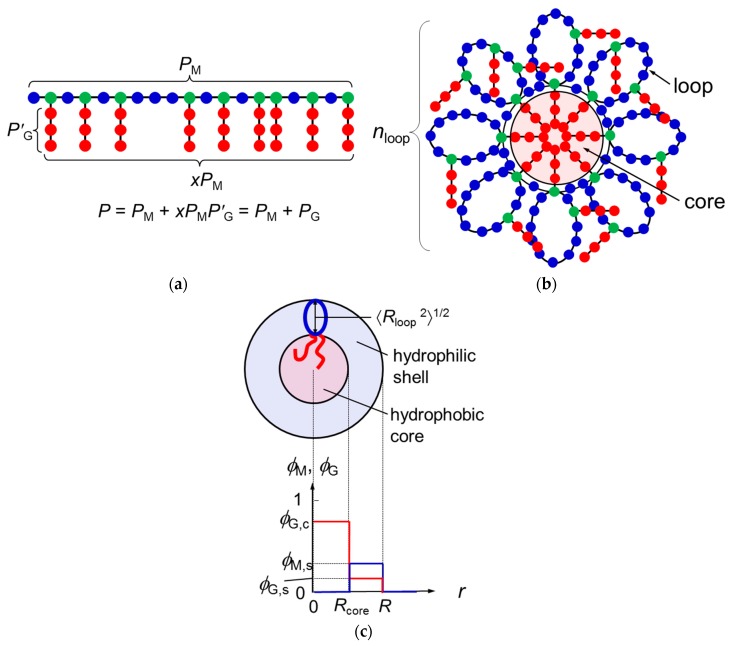
Schematic diagrams of the graft copolymer chain (**a**), the flower micelle formed by the copolymer chain (**b**), and the radial concentration profiles of the main-chain and graft-chain units in the flower micelle (**c**). In Panel b, the flower micelle is constructed by *m* copolymer chains. In Panel a, blue and green circles are referred to as the main chain, and red circles as the graft chain. In Panels a and b, blue circles are called the A unit, and red and green circles as the B unit to discuss the mixing enthalpy (cf. Equations (9)–(11)).

Let us consider the graft copolymer illustrated in Figure 1a. The main chain and graft chains consist of *P*_M_ units and *P*′_G_ units, respectively. The mole fraction of the branch units on the main chain is denoted as *x*, and the distribution of the branch units along the main chain is assumed to be random or periodic (not block-like). The total number of the graft-chain units per copolymer chain is *P*_G_ = *xP*_M_*P*′_G_, and the total degree of polymerization of the graft copolymer chain is *P* = *P*_M_ + *P*_G_ = *P*_M_(1 + *xP*′_G_). It is assumed that the main-chain and graft-chain units as well as the solvent S molecule occupy lattice sites with a common size *a*.

If the graft-chain unit is sufficiently hydrophobic, graft chains of the copolymer tend to aggregate to form a hydrophobic core, and the main chain tends to form loop chains in aqueous medium. As a result, the *m* copolymer chains construct a flower micelle illustrated in Figure 1b; *m* is the copolymer-chain aggregation number of the micelle. Only graft chains attaching to roots of the loops can enter the hydrophobic core, and the remaining graft chains are outside the core.

According to the wormlike chain model [13,14,15], the ring closure probability of the chain rapidly diminishes to zero at the chain contour length reducing to ca. 1.6*q*, where *q* is the persistence length. This means that the main chain portion shorter than 1.6*q* cannot form the loop because of the chain stiffness. In what follows, we consider the flower micelle consisting of loop chains with this “minimum loop size” [6]. The number of main-chain units per the minimum loop chain *P*_loop_, and the number of loop chains per chain *n*_loop_ are calculated by

(1) $$P_{\mathrm{loop}}=\frac{1.6q}{a},n_{\mathrm{loop}}=\frac{P_{M}}{P_{\mathrm{loop}}}$$

The numbers of graft chains included in the hydrophobic core and outside of the core, *x*_c_*P*_M_ and *x*_s_*P*_M_, respectively, are calculated by

(2) $$x_{c}P_{M}=\lambda n_{\mathrm{loop}},x_{s}=x-x_{c}$$

where *λ* is the number of side chains included in the core at each root of the loop. (Figure 1b illustrates the case of *λ* = 1). It has been assumed in Equation (2) that *n*_loop_ is much larger than unity.

In the previous paper [12], we regarded the spherical micelle formed by di-block copolymer chains as a thermodynamic phase, assuming that the aggregation number of the micelle is sufficiently large. Similarly, the present study regards the flower micelle as a thermodynamic phase to demonstrate the micellization of the graft copolymer in a selective solvent. Furthermore, we use a simple model for the flower micellar phase, of which radial concentration profiles (volume fractions) of the main-chain and graft-chain units are given by

(3) $$\phi_{M}=\left\{ \begin{array}{ll} 0, & 0\leq r<R_{\mathrm{core}}\\ \phi_{M,s}, & R_{\mathrm{core}}\leq r<R\\ 0, & R\leq r \end{array}\right.,\;\phi_{G}=\left\{ \begin{array}{ll} \phi_{G,c}, & 0\leq r<R_{\mathrm{core}}\\ \phi_{G,s}, & R_{\mathrm{core}}\leq r<R\\ 0, & R\leq r \end{array}\right.$$

(cf. Figure 1c). Here, *R*_core_ and *R* are the radii of the micelle core and the whole micelle, respectively, and the solvent volume fraction is given by ϕ_S_ = 1 − ϕ_M_ − ϕ_G_ at each radial distance *r*. Furthermore, using the wormlike chain model, *R*_core_^2^ and the mean square distance from the end to the midpoint of the loop 〈*R*_loop_^2^〉 (cf. Figure 1c) are expressed in terms of the persistence lengths of the graft chain (*q*_G_) and of the copolymer main chain (*q*), respectively, by [15]

(4) $${\left(R_{\mathrm{core}}/a\right)}^{2}=\left(2q_{G}/a\right){P^{\prime }}_{G}-2{\left(q_{G}/a\right)}^{2}\left(1-e^{-{P^{\prime }}_{G}a/q_{G}}\right)$$

(5) $$\frac{\langle {R_{\mathrm{loop}}}^{2}\rangle }{a^{2}}=\frac{P_{\mathrm{loop}}{}^{2}}{\sqrt{42.5-\frac{32}{3}\left(aP_{\mathrm{loop}}/2q\right)+16{\left(aP_{\mathrm{loop}}/2q\right)}^{2}}}$$

(cf. Appendix A). The radius *R* of the whole micelle is calculated by

(6) $$R=R_{\mathrm{core}}+{\langle {R_{\mathrm{loop}}}^{2}\rangle }^{1/2}$$

The average volume fraction *ϕ*_P_ of the copolymer in the flower micelle phase is given by

(7) $$\phi_{P}=\frac{3a^{3}Pm}{4\pi R^{3}}$$

and the volume fractions ϕ_M,s_, ϕ_G,s_, and ϕ_G,c_ are related to ϕ_P_ by

(8) $$\phi_{M,s}=\frac{R^{3}P_{M}\phi_{P}}{\left(R^{3}-R_{\mathrm{core}}{}^{3}\right)P},\phi_{G,s}=\frac{R^{3}x_{s}P_{M}{P^{\prime }}_{G}}{\left(R^{3}-R_{\mathrm{core}}{}^{3}\right)P}\phi_{P},\phi_{G,c}=\frac{R^{3}x_{c}P_{M}{P^{\prime }}_{G}}{R_{\mathrm{core}}{}^{3}P}\phi_{P}$$

From the last equation for ϕ_G,c_ in Equation (8), it can be seen that ϕ_P_ must be equal to or less than *PR*_core_^3^*/x*_c_*P*_M_*P*′_G_*R*^3^, because ϕ_G,c_ does not exceed unity. Furthermore, since *m* must be larger than unity, *P*_M_ must be smaller than 4π*R*^3^ϕ_P_/3*a*^3^(1 + *xP*′_G_) from Equation (7).

For amphiphilic random or periodic copolymers, the ionizable group or hydrophilic side-chain group of each hydrophilic monomer unit is substituted by the hydrophobic graft chain. Thus, the branch unit in the main chain (green circles in Figure 1a,b) may be hydrophobic, having interaction parameters much different from those of the non-branch unit (i.e., the hydrophilic monomer unit) in the main chain but similar to those of the graft-chain unit. We refer to the non-branch unit in the main chain as the A unit and to the graft-chain unit as well as the branch unit in the main chain as the B unit, neglecting the difference in the interaction between the graft-chain unit and the branch unit in the main chain. The volume fractions of the A and B units in the shell and core phases are given by

(9) $$\phi_{A,s}=\frac{R^{3}\left(1-x\right)P_{M}}{\left(R^{3}-R_{\mathrm{core}}{}^{3}\right)P}\phi_{P},\phi_{B,s}=\frac{R^{3}\left(x+x_{s}{P^{\prime }}_{G}\right)P_{M}}{\left(R^{3}-R_{\mathrm{core}}{}^{3}\right)P}\phi_{P},\phi_{B,c}=\frac{R^{3}x_{c}{P^{\prime }}_{G}P_{M}}{R_{\mathrm{core}}{}^{3}P}\phi_{P}$$

and the mole fractions of the A and B units in the copolymer chain are written as

(10) $$x_{A}=\left(1-x\right)\frac{P_{M}}{P},x_{B,s}=\left(x+x_{s}{P^{\prime }}_{G}\right)\frac{P_{M}}{P},x_{B,c}=x_{c}{P^{\prime }}_{G}\frac{P_{M}}{P},x_{A}+x_{B,s}+x_{B,c}=1$$

where *x*_B,s_ and *x*_B,c_ are the mole fractions of the B unit in the shell and core regions, respectively. The solvent volume fractions in the shell and core regions are given by ϕ_S,s_ = 1 − ϕ_A,s_ − ϕ_B,s_ and ϕ_S,c_ = 1 − ϕ_B,c_, respectively.

We apply the Flory–Huggins theory [16] to the flower micelle phase to formulate the mixing Gibbs energy per lattice site Δ*g*_m_ of the micelle phase, which consists of the mixing entropy Δ*S*, the mixing enthalpy Δ*H*, and the interfacial Gibbs energy 4π*R*_core_^2^*γ* (*γ*: the interfacial tension between the core and shell regions of the micelle). The formulation method is described in Appendix B. The final result is written as

(11) $$\begin{array}{ll} \frac{\Delta g_{m}}{k_{B}T} & =\left(\frac{-T\Delta S+\Delta H+4\pi R_{\mathrm{core}}{}^{2}\gamma }{k_{B}T}\right)/\frac{4\pi R^{3}}{3a^{3}}\\ & =\frac{\phi_{P}}{P}\ln\left(\kappa \phi_{P}\right)+\frac{R^{3}-R_{c}{}^{3}}{R^{3}}\phi_{S,s}\ln\phi_{S,s}+\frac{R_{c}{}^{3}}{R^{3}}\phi_{S,c}\ln\phi_{S,c}\\ & +\left[x_{A}\phi_{S,s}\chi_{\mathrm{AS}}+\left(x_{B,s}\phi_{S,s}+x_{B,c}\phi_{S,c}\right)\chi_{\mathrm{BS}}-x_{A}\left(x_{B,s}+x_{B,c}-\phi_{B,s}\right)\chi_{\mathrm{AB}}\right]\phi_{P}\\ & +\frac{3{\left(R_{\mathrm{core}}/a\right)}^{2}}{{\left(R/a\right)}^{3}}\frac{a^{2}}{k_{B}T}\gamma \end{array}$$

where *χ*_AS_, *χ*_BS_, and *χ*_AB_ are the interaction parameters between S and A, between S and B, and between A and B, respectively, *κ* is defined by Equation (B11), and (*a*^2^*/k*_B_*T*)*γ* is calculated by Equation (B13) with Equation (B14). The term ln *κ* includes the conformational entropy loss at the formation of the flower micelle.

When the graft copolymer solution is homogeneous, the mixing Gibbs energy per lattice site Δ*g*_h_ is given by [16]

(12) $$\frac{\Delta g_{h}}{k_{B}T}=\left(1-\phi_{P}\right)\ln\left(1-\phi_{P}\right)+\frac{\phi_{P}}{P}\ln\phi_{P}+\bar{\chi }\left(1-\phi_{P}\right)\phi_{P}$$

with the average interaction parameter $\bar{\chi }$ between the graft copolymer chain and solvent, defined by [17]

(13) $$\bar{\chi }\equiv x_{A}\chi_{\mathrm{AS}}+\left(1-x_{A}\right)\chi_{\mathrm{BS}}-x_{A}\left(1-x_{A}\right)\chi_{\mathrm{AB}}.$$

## 3. Results and Discussion

Because we did not consider above the interaction among flower micellar phases in the solution, the following discussion is limited to dilute solutions of random and periodic copolymers. Ueda et al. [9] reported the molecular weight dependence of the micellization behavior for the amphiphilic alternating copolymer of sodium maleate and dodecyl vinyl ether, P(MAL/C12), in dilute aqueous solutions including 0.05 M NaCl. First, we examine theoretically the micellization behavior of an alternating copolymer mimicking P(MAL/C12).

In the lattice theory, the choice of the unit lattice site is rather arbitrary. Here, we assume the main-chain portion (the C_2_ unit) of maleate or dodecyl vinyl ether monomer unit is chosen as the unit lattice site. Then, the hydrophobic dodecyl side chain is assumed to occupy six lattice sites, i.e., *P*′_G_ = 6. (The carboxy group and the ether oxygen atom in the maleate and dodecyl vinyl ether monomer units are not considered explicitly; they are assumed to be included in the main-chain portions). In aqueous solutions, a strong electrostatic repulsion acts among maleate units (the A unit), while a hydrophobic attraction acts among the C_2_ units of the dodecyl group (the B unit). The strong electrostatic repulsion and hydrophobic attraction are expressed using a negative *χ*_AS_ and positive *χ*_BS_, respectively. (To account for the long range electrostatic interaction, the unit lattice site may have to be larger than the C_2_ unit, but the following results do not essentially change by the choice of the unit lattice site). Since we here focus on the amphiphilicity of the graft copolymer, we assume *χ*_AB_ to be zero, as in the previous study [12]. (The change of the *χ*_AB_ value may be compensated by adjusting values of *χ*_AS_ and *χ*_BS_).

**Figure 2:**
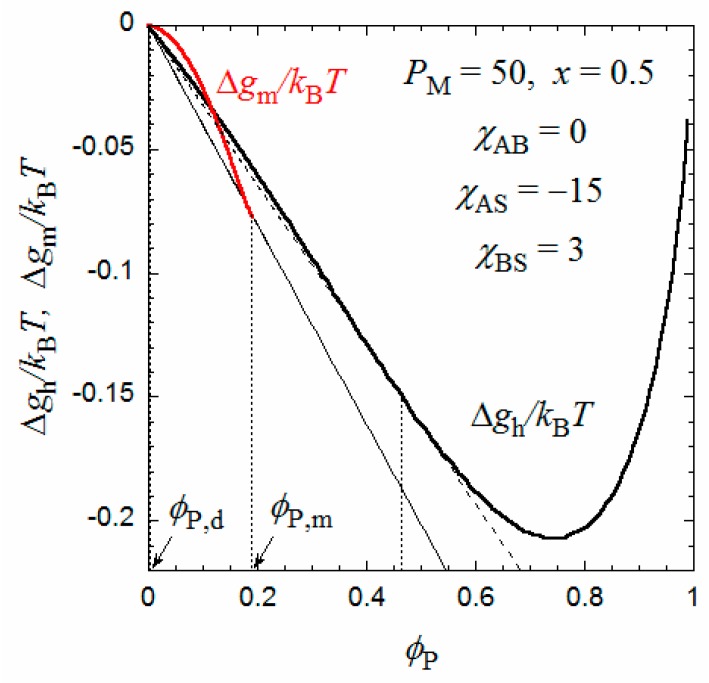
Concentration dependences of Δ*g*_m_ and Δ*g*_h_ at *x* = 0.5 calculated by Equations (9) and (10).

Figure 2 shows the copolymer concentration dependences of Δ*g*_m_ (red curve) and Δ*g*_h_ (black curve) calculated by Equations (11) and (12). We have chosen *P*_M_ = 50, *x* = 0.5, *χ*_AS_ = −15, *χ*_BS_ = 3, and *χ*_AB_ = 0 ($\bar{\chi }$ = 0.75). All remaining parameters included in Equation (11) can be calculated from *a* = 0.25 nm (the contour length per the main-chain monomer (C_2_) units), and *q* = 3 nm, *q*_G_ = 0.53 nm, and *λ* = 3 determined previously [9]. We can draw a common tangent (the thin line) to the dilute side of the black curve and red curve. (It is seen that the black curve has a downward convex shape around ϕ_P_ = 0, if it is enlarged). The copolymer volume fractions at the two points of contact of the common tangent, denoted as ϕ_P,d_ and ϕ_P,m_, are binodal concentrations of the coexisting dilute and micellar phases, respectively. The tangent line is below the common tangent line (the thin broken line) for the thick black curve for Δ*g*_h_, indicating that the micellization is thermodynamically more stable than the phase separation into two homogeneous phases.

Similar curves for Δ*g*_m_ and Δ*g*_h_ were obtained for different *P*_M_, and the volume fraction ϕ_P,m_ of the equilibrium micellar phase were determined by the above method. The aggregation number *m* of the copolymer chains per micelle can be calculated from Equation (7), i.e.,

(14) $$m=\frac{4\pi R^{3}}{3a^{3}P}\phi_{P,m}$$

**Figure 3:**
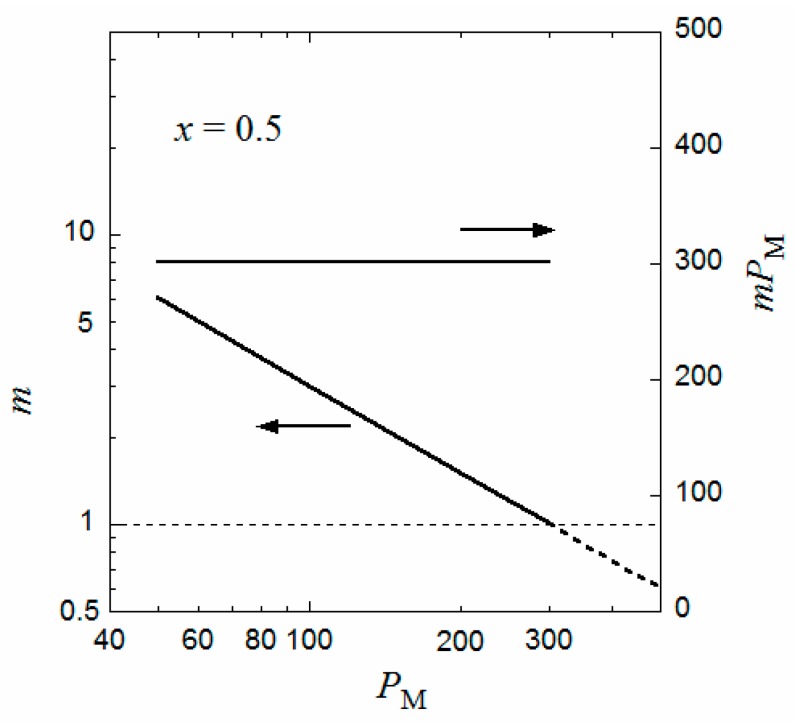
Degree of polymerization dependences of the aggregation number *m* and *mP*_M_ at the interaction parameters identical to those in Figure 2.

Figure 3 shows the degree of polymerization *P*_M_ dependence of *m* such obtained as well as the product *mP*_M_ (the number of monomer units per micelle) at the interaction parameters identical to those in Figure 2. It is seen that *m* is inversely proportional to *P*_M_, and the product *mP*_M_ is independent of *P*_M_. (Because *P* is proportional to *P*_M_ and *R* is independent of *P*_M_, the inverse proportionality of *m* to *P*_M_ comes from the *P*_M_ independence of ϕ_P,m_ calculated from the comparison between of the Δ*g*_m_ and Δ*g*_h_ curves). This relation was observed experimentally for P(MAL/C12) in 0.05 M aqueous NaCl solution [9] as well as for a random copolymer of poly(ethylene glycol) methyl ether methacrylate and dodecyl methacrylate, P(PEGMA/DMA), in water [18]; however, for P(PEGMA/DMA) with *x* = 0.5, the constant *mP*_M_ is slightly larger than 300. The value of *mP*_M_ changes by values of *q*, *λ*, and the interaction parameters. It is noted that the formulation of Δ*g*_m_ in the previous section can apply both to periodic and random copolymers.

When *P*_M_ approaches 300 in Figure 3, *m* tends to unity, and ϕ_P,d_ corresponding to the critical micelle concentration (cmc) of the coexisting dilute phase becomes very low (not shown). That is, when *P*_M_ approaches 300, the flower micelle is formed by one copolymer chain (the unimer micelle), and the cmc tends to zero. This situation resembles the liquid–liquid phase separation in a homopolymer polymer solution with an infinitely high-molecular-weight polymer, where the polymer volume fraction at the critical point is predicted to be zero by the conventional Flory–Huggins theory [16].

When the same calculation of *m* is performed where *P*_M_ > 300, the inverse proportionality of *m* to *P*_M_ still holds even if *P*_M_ exceeds 300, as indicated by the dashed line in Figure 3. However, because the aggregation number is less than unity, some portion of the main chain is not included in the flower micelle at *P*_M_ > 300. For example, at *P*_M_ = 600 where *m* = 0.5, half of the main chain is not included in the flower micelle. This half main-chain portion may form another flower micelle. As a result, the whole copolymer chain forms a double-core flower necklace. (Strictly speaking, the double-core flower necklace needs a bridge chain connecting two unit flowers, so that *P*_M_ must be slightly larger than 600 to form the double-core flower necklace). In fact, Ueda et al. [9] reported the transition from the flower micelle to the flower necklace at *P*_M_ exceeding 300.

The flower micelle is formed also by amphiphilic random copolymers with hydrophobic dodecyl side chains of *x* < 0.5 in aqueous solutions. Next, we examine the hydrophobic monomer content dependence of the micellization for an aqueous solution of an amphiphilic random copolymer, calculated in the same way from the Δ*g*_m_ and Δ*g*_h_ curves as in Figure 2. The number *λ* of side chains included in the core at each root of the loop appearing in Equation (2) may be dependent on the monomer content *x*. In the limit of *x* = 1/*P*_loop_, each loop chain has only one hydrophobic side chain on average. Thus, *λ* = 1 at *x* = 1/*P*_loop_. When *x* increases, *λ* may first increase from unity and approach an asymptotic value. For a given value of *λ*, ϕ_P,d_ and ϕ_P,m_ of the coexisting dilute and micellar phases can be calculated as functions of *x* from the curves of Δ*g*_m_ and Δ*g*_h_ as mentioned above.

**Figure 4:**
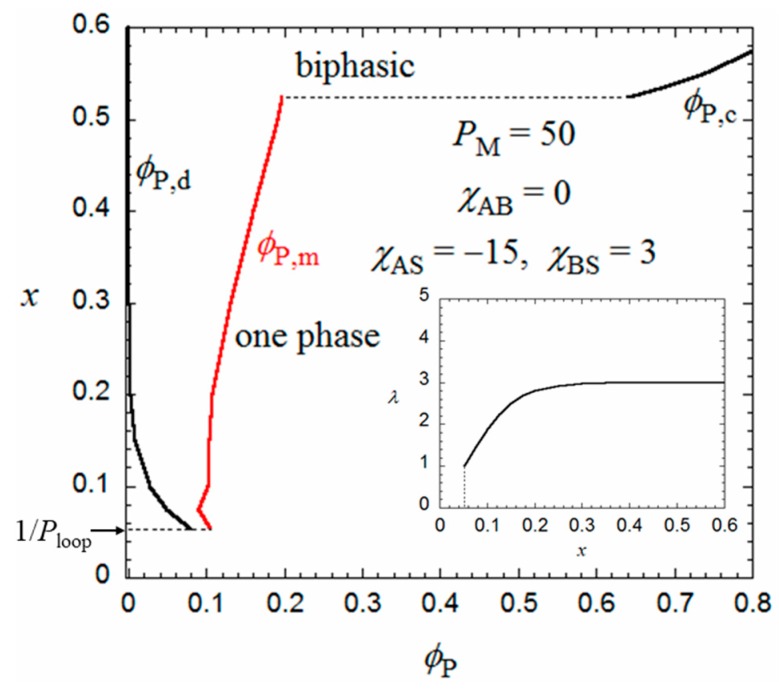
Monomer content-concentration phase diagram for an aqueous solution of a random copolymer with *P*_M_ = 50 and the same interaction parameters as those used in Figure 2 and Figure 3.

Figure 4 shows *ϕ*_P,d_ and *ϕ*_P,m_ obtained for the amphiphilic random copolymer with a *P*_M_ of 50 and the same interaction parameters used in Figure 2 and Figure 3, in the *x*-ϕ_P_ phase diagram. The *x* dependence of *λ* used is shown in the insert of Figure 4. When *x* is decreased from 0.5, ϕ_P,d_ (cmc) increases, and the copolymer in a dilute solution (ϕ_P_ < 0.08) transforms from the flower micelle to the random coil at passing the bimodal curve for ϕ_P,d_ (cmc). At *x* < 1/*P*_loop_, the loop size of the flower micelle should be larger than the minimum size given by Equation (1). We do not discuss such a loose flower micelle here.

**Figure 5:**
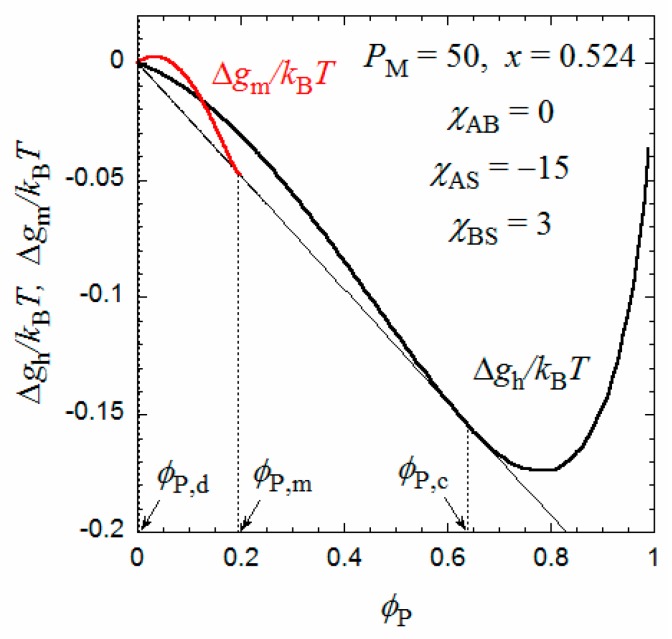
Concentration dependences of Δ*g*_m_ and Δ*g*_h_ at *x* = 0.524 calculated by Equations (9) and (10).

On the other hand, when *x* increases from 0.5, the Δ*g*_h_ − ϕ_P_ curve goes down relative to the Δ*g*_m_ − ϕ_P_ curve, and as shown in Figure 5, at *x* = 0.524, we can draw a common tangent (the thin line) to the dilute and concentrated sides of the black curve (Δ*g*_h_) and the red curve (Δ*g*_m_). When *x* > 0.524, the phase separation into dilute and concentrated homogeneous phases with concentrations ϕ_P,d_ and ϕ_P,c_ becomes thermodynamically more stable than the micellization. As a result, the phase gap in the *x*-ϕ_P_ phase diagram is abruptly enlarged when *x* > 0.524, as shown in Figure 4. To the best of my knowledge, there have hitherto been no reports of the corresponding crossover from micellization to liquid–liquid phase separation as the hydrophobic content *x* increases.

Eisenberg et al. [19] investigated the random copolymer of styrene and methacrylic acid with *x* ~ 0.8, which was first dissolved in dioxane or tetrahydrofuran (THF), followed by the addition of water, and observed “large compound micelles” and “bowl-shaped aggregates” by transmission electronic microscopy (TEM). Here, the “large compound micelle” is the large homogeneous polymer-rich spheres, corresponding to the droplet of the concentrated homogeneous phase formed by the liquid–liquid phase separation, predicted in Figure 4, and a “bowl-shaped aggregate” may be formed from the concentrated-phase droplet in which solvent bubbles are trapped [19]. Wang et al. [20] reported the formation of uniform colloidal spheres by an amphiphilic random copolymer, poly{2-[4-(phenylazo)phenoxy]ethyl acrylate-*co*-acrylic acid}, where *x* = 0.5 in THF–water mixtures with high water concentrations. This may be another example of the liquid–liquid phase separation of the amphiphilic random copolymer in solution. Zhang et al. [21] studied the self-association of amphiphilic graft (periodic) copolymers in a hypothetical solution of the two-dimensional space by the self-consistent field theory. Although they assumed perfect flexibility and comparable chain lengths of the main and graft chains, being different conditions from the present study, they observed a “large compound micelle” at higher graft density (i.e., higher hydrophobic content *x*) under weaker amphiphilicity (cf. Figure 8a in [21], where the graft chain number = 5).

Yusa et al. [22] found a transition from the unimer micelle to the single random coil chain of a random copolymer of hydrophilic sodium 2-(acrylamido)-2-methylpropanesulfonate and hydrophobic 11-acrylamidoundecanoic acid (AmU) where *x* = 0.5 in 0.1 M aqueous NaCl solution by changing pH. At pH = 3, where the carboxy group is not ionized, AmU was strongly hydrophobic, and the copolymer formed a unimer micelle with *m* = 1. The degree of polymerization *P*_M_ of the copolymer sample (= 475) was slightly larger than 300 (cf. Figure 3), maybe due to the difference in the parameters, e.g., *P*’_G_ and *λ*, from those used in Figure 3. On the other hand, at pH = 9, where the carboxy group of AmU is ionized, the copolymer was transformed to a random coil. In Figure 2, the Δ*g*_m_ curve goes up and the Δ*g*_h_ has no inflection point when *χ*_BS_ is decreased from 3, i.e., AmU becomes more hydrophilic. As a result, the random coil conformation in the homogeneous phase becomes more stable than the flower micelle, which is consistent with Yusa et al.’s finding. The transition from the unimer micelle to the single random coil chain by decreasing *x*, predicted in Figure 4, was reported by Fujimoto and Sato [11].

Recently, several authors have reported that amphiphilic random copolymers form vesicles in dilute solutions [23,24,25,26], which was not considered in the present study. Zhu and Liu [24] investigated vinyl polymers bearing L-glutamic acid moieties and dodecyl groups in the random sequence to find the vesicle in water at a high hydrophobic content *x* (>0.75). Their random copolymer samples possess low degrees of polymerization (<36). For these samples to form the flower micelle, *n*_loop_ should be less than 2 and one loop chain should bear many hydrophobes. The present theory may not be able to be applied to such random copolymers.

Tian et al. [25] observed vesicles as well as hollow tubes and wormlike rods formed by poly(hydroxyethyl methacrylate) (PHEMA) partially and randomly modified by the hydrophobic 2-diazo-1,2-naphthoquinone in solution. These copolymer samples were dissolved in dimethyl- formamide, followed by the addition of water, and finally dialyzed against water to form the vesicle. Because even PHEMA is insoluble in water, the vesicle formed must not be in the thermodynamically stable state, which cannot be treated in the present statistical thermodynamic theory.

Ghosh et al. [26] reported that an amphiphilic random copolymer of hydrophilic tri(oxyethylene) methacrylamide and hydrophobic *n*-octyl methacrylate exhibited a thermally induced vesicle to spherical micelle transition. However, it should be noted that the illustration of the spherical micelle by these authors (cf. Scheme 1 in [26]) was inconsistent with the experimental TEM observation of spherical aggregates (diameter in the range of 70–80 nm) at 60 °C. In the illustration, the hydrophilic and hydrophobic side chains were in the coronal and core regions of the micelle, respectively, and the whole copolymer main chain was confined to the corona-core interface. If this is the case, the diameter of the micelle must be equal to twice the sum of the hydrophilic and hydrophobic side chain lengths. Even if the side chains are fully extended, such an estimated diameter is as small as 6 nm, which is much smaller than the diameter of the spherical aggregate at 60 °C. Thus, the spherical aggregate at 60 °C may not be the spherical micelle indicated in their illustration, but the phase-separated concentrated phase droplet, because both kinds of side chains are hydrophobic at 60 °C above the lower critical solution temperature [26].

## 4. Conclusions

The flower micelle formed by amphiphilic random and periodic copolymers in solution was regarded as a thermodynamic phase to formulate the mixing Gibbs energy. The formulated mixing Gibbs energy of the micelle was compared with that of the homogeneous phase to calculate (1) the aggregation number *m* of the micelle as a function of the degree of polymerization *P*_M_ of the copolymer chain, (2) the cmc as a function of the hydrophobic content *x*, and (3) the crossover *x* from micellization to liquid–liquid phase separation. 

The above theoretical results were compared with experimental results for amphiphilic random and alternating copolymers reported previously. Prediction (1) was confirmed experimentally [9,18], and the experimentally observed transition from the uni-core flower micelle to the multi-core flower necklace [9] was also consistent with the present theory. The “large compound micelle” previously observed for amphiphilic random copolymers [19,20] may correspond to the concentrated-phase droplets produced by liquid–liquid phase separation, which was predicted to occur in this theory. The transition from the unimer micelle to the single random coil chain [11,22] was also predicted by this theory.

The limitation of the present theory was also discussed. The present theory may not be able to be applied to amphiphilic random copolymers of low degrees of polymerization and high hydrophobic contents [24], nor to frozen micelles that are not in a thermodynamic equilibrium state [25].

## Appendix A. Mean Square Distance from the End to the Midpoint of the Loop

The mean square distance from the end to the midpoint of the loop 〈*R*_loop_〉 (cf. Figure 1c) near the rod and coil limits is calculated using the wormlike chain model. Yamakawa and Stockmayer [13] formulated 〈*R*_loop_^2^〉 for the wormlike chain near the rod limit. Their result is written as

(A1) $$\langle {R_{\mathrm{loop}}}^{2}\rangle ={\left[2q\left(I_{3}/2I_{2}\right)N_{K}\right]}^{2}\left(N_{K}<<1\right)$$

where *q* and *N*_K_ is the persistence length and the Kuhn statistical segment number, respectively, and *I*_2_ and *I*_3_ are calculated by

(A2) $$I_{2}\equiv \int_{\theta /2}^{\pi }\frac{1}{\sqrt{C-\cos\omega }}d\omega ,I_{3}\equiv \frac{1}{2I_{2}}\int_{\theta /2}^{\pi }\frac{\sin\omega }{\sqrt{C-\cos\omega }}d\omega$$

with the angle *θ* formed by the tangent vectors at both chain ends and a constant *C* determined by the equation

(A3) $$I_{1}\equiv \int_{\theta /2}^{\pi }\frac{\cos\omega }{\sqrt{C-\cos\omega }}d\omega =0$$

Near the rod limit, the energetically most stable loop conformation gives us the results, *θ* = 1.7208, *C* = 0.6522, *I*_2_ = 3.29, and *I*_3_ = 2.58.

Using the first Daniels approximation, we can calculate 〈*R*_loop_^2^〉 near the coil limit as [27]

(A4) $$\langle {R_{\mathrm{loop}}}^{2}\rangle ={\left(2q\right)}^{2}\left(\frac{1}{4}N_{K}+\frac{1}{12}\right)\left(N_{K}>>1\right)$$

Equation (5) in the text is the interpolation of 〈*R*_loop_^2^〉 given by Equations (A1) and (A4) near rod and coil limits by use of the Padé approximation.

## Appendix B. Mixing the Gibbs Energy of the Flower Micelle Phase

To calculate the mixing entropy of the flower micelle phase, we counted the number Ω of arrangements of *m* graft copolymer chains into the concentric spherical lattice with the inner and outer spherical radii *R*_core_ and *R*, respectively, shown in Figure 1c in the text. Each main chain of the graft copolymer may form loops, trains, and tails on the core–shell interface, but we assume that both hydrophilicity of the main chain and hydrophobicity of the graft-chain are so strong that both train and tail chains are negligibly short.

The first unit of the main chain in the first copolymer chain must be located at one of the lattice sites on the core–shell interface. The number of such lattice sites is given by *N*_intf_ = 4π (*R*_core_*/a*)^2^. The number *ω*^′^*_i_* of lattice sites where the first unit of the *i*-th copolymer chain is given by

(B1) $${\omega^{\prime }}_{i}=N_{\mathrm{intf}}{f^{\prime }}_{i-1},{f^{\prime }}_{i-1}=1-\frac{n_{\mathrm{loop}}}{N_{\mathrm{intf}}}\left(i-1\right)\left(1\leq i\leq m\right)$$

where *f*’*_i_*_−1_ is the probability of the vacancy for the lattice site on the core–shell interface when first units of *i* – 1 copolymer chains have been already arranged.

The flower micelle contains *mn*_loop_ loop chains and *mx*_s_*P*_M_ graft chains in the shell region. The first unit of the first copolymer chain is identical with the first unit of the first loop chain, and the last unit of the first loop chain must be located in the neighboring site of the first one of the same loop chain on the core–shell interface. Furthermore, the loop chain cannot be located in the core region, i.e., the core–shell interface acts as a reflecting barrier. The first loop chain possesses *x*_s_*P*_loop_ graft chains with the degree of polymerization *P*’_G_. Thus, the number of arrangements *ω*_s,1_ of the first loop chain is given by [12,28]

(B2) $$\omega_{s,1}=\frac{{\left(z-1\right)}^{P_{\mathrm{loop}}}}{\left(\sqrt{\pi }/3\right)P_{\mathrm{loop}}}G(0;aP_{\mathrm{loop}}/2q){\left(z-1\right)}^{x_{s}P_{\mathrm{loop}}{P^{\prime }}_{G}}$$

where *G*(0,*aP*_loop_/2*q*) is the ring closure probability. Shimada and Yamakawa [14] proposed an expression of the probability for the wormlike chain as

(B3) $$G(0;aP_{\mathrm{loop}}/2q)=\frac{28.01}{{\left(aP_{\mathrm{loop}}/2q\right)}^{5}}\exp\left[-\frac{7.027}{aP_{\mathrm{loop}}/2q}+0.492\left(aP_{\mathrm{loop}}/2q\right)\right]$$

Similarly, the number of arrangements *ω*_s,*i*_ of the *i*-th loop chain is given by

(B4) $$\omega_{s,j}=\omega_{s,1}f_{s,j-1}{}^{P_{\mathrm{loop}}\left(1+x_{s}{P^{\prime }}_{G}\right)},f_{s,j-1}=1-\frac{\phi_{M,s}+\phi_{G,s}}{mn_{\mathrm{loop}}}(j-1)\left(1\leq j\leq mn_{\mathrm{loop}}\right)$$

Here, *f*_s,*j*−1_ is the probability of the vacancy for the lattice site in the shell region when *j* – 1 loop chains have been already arranged. The core region of the flower micelle consists of *mx*_c_*P*_M_ graft chains. Numbers of arrangements *ω*_c,1_ and *ω*_c,*i*_ of the first and *i*-th graft chains in the core region are written as

(B5) $$\omega_{c,1}=\frac{{\left(z-1\right)}^{{P^{\prime }}_{G}-1}}{\left(\sqrt{\pi }/3\right){P^{\prime }}_{G}},\;\omega_{c,k}=\omega_{c,1}f_{c,k-1}{}^{{P^{\prime }}_{G}},f_{c,k-1}=1-\frac{\phi_{G,c}}{\lambda mn_{\mathrm{loop}}}(k-1)\left(1\leq k\leq \lambda mn_{\mathrm{loop}}\right)$$

where *f*_c,*k*−1_ is the probability of the vacancy for the lattice site in the core region when *k* − 1 graft chains have been already arranged.

Using the above results, the number of arrangements Ω of the total *m* graft copolymer chains on the concentric spherical lattice is calculated by

(B6) $$\Omega =\prod_{i=1}^{m}{\omega^{\prime }}_{i}\prod_{j=1}^{mn_{\mathrm{loop}}}\omega_{s,j}\prod_{k=1}^{\lambda mn_{\mathrm{loop}}}\omega_{c,k}$$

or

(B7) $$\ln\Omega =m\ln\left[N_{\mathrm{intf}}{\left(\omega_{s,1}\omega_{c,1}{}^{\lambda }\right)}^{n_{\mathrm{loop}}}\right]+\sum_{i=1}^{m}\ln{f^{\prime }}_{i-1}+\sum_{j=1}^{mn_{\mathrm{loop}}}\ln\left[f_{s,j-1}{}^{P_{\mathrm{loop}}\left(1+x_{s}{P^{\prime }}_{G}\right)}f_{c,j-1}{}^{\lambda {P^{\prime }}_{G}}\right]$$

The numbers of arrangements of the uniform bulk copolymer (Ω_P_) and the bulk solvent (Ω_S_) are given respectively by [16]

(B8) $$\ln\Omega_{P}=m\ln\left(mP\omega_{P,1}{}^{n_{\mathrm{loop}}}\right)+\sum_{i=1}^{m}\ln{f^{\prime }}_{P,i-1}+\sum_{j=1}^{mn_{\mathrm{loop}}}\ln f_{P,j-1},\ln\Omega_{S}=0$$

where

(B9) $$\omega_{P,1}={\left(z-1\right)}^{P_{\mathrm{loop}}\left(1+x{P^{\prime }}_{G}\right)},{f^{\prime }}_{P,i-1}=1-\frac{i-1}{m},f_{P,j-1}=1-\frac{j-1}{mn_{\mathrm{loop}}}$$

Therefore, the entropy of mixing Δ*S* in the micellar phase is given by

(B10) $$\begin{array}{ll} \frac{\Delta S}{mk_{B}} & =\frac{1}{m}\left(\ln\Omega -\ln\Omega_{P}-\ln\Omega_{S}\right)\\ & =-\ln\kappa -\ln\phi_{P}-n_{\mathrm{loop}}\left[P_{\mathrm{loop}}\left(1+x_{s}{P^{\prime }}_{G}\right)\frac{\phi_{S,s}}{1-\phi_{S,s}}\ln\phi_{S,s}+\lambda {P^{\prime }}_{G}\frac{\phi_{S,c}}{1-\phi_{S,c}}\ln\phi_{S,c}\right] \end{array}$$

where ϕ_S_ = 1 − ϕ_M_ − ϕ_G_, *k*_B_ is the Boltzmann constant, and *κ* is defined by

(B11) $$\kappa \equiv \frac{{\left(R/a\right)}^{3}}{3{\left(R_{\mathrm{core}}/a\right)}^{2}}{\left[\frac{\pi P_{\mathrm{loop}}{P^{\prime }}_{G}{}^{\lambda }}{9G(0;{\tilde{l}}_{\mathrm{loop}})}\right]}^{n_{\mathrm{loop}}}$$

To obtain Equation (B10), we used Equation (5) in the text, approximated *f*’*_i_*_−1_ ≈ *f*’_P,*i*__−1_, and replaced the summations with respect to *j* in Equations (B7) and (B8) by integrations on the assumption of *mn*_loop_ >> 1. The parameter *κ* can be calculated from *P*’_G_, *P*_loop_, and 2*q/a* using Equations (3), (4) and (B3).

At calculating the mixing enthalpy Δ*H* of the micelle phase and the interfacial tension *γ* between the core and shell regions of the flower micelle, we assume that the branch units in the main chain have the same interaction parameters as those of the graft-chain units. In what follows, the non-branch unit in the main chain is referred to as the A unit, and the graft-chain unit as well as the branch unit in the main chain are referred to as the B unit. Under the mean-field approximation [16], Δ*H* is given by

(B12) $$\frac{\Delta H}{mPk_{B}T}=x_{A}\phi_{S,s}\chi_{\mathrm{AS}}+\left(x_{B,s}\phi_{S,s}+x_{B,c}\phi_{S,c}\right)\chi_{\mathrm{BS}}-x_{A}\left(x_{B,s}+x_{B,c}-\phi_{B,s}\right)\chi_{\mathrm{AB}}$$

where *χ_αβ_* (*α*, *β* = S, A, B) is the interaction parameter between species *α* and *β* (S stands for the solvent; the definition of *χ_αβ_* is slightly different from that of [16]), and *x*_A_, *x*_B,s_, and *x*_B,c_ are the mole fractions of the A and B units (existing in the shell and core regions) defined by Equation (8).

Noolandi and Hong [17] formulated the interfacial tension *γ* between the core and shell regions of the spherical micelle. We may extend their result to *γ* for the flower micelle, where the asymptotic volume fractions of the A and B units are ϕ_A,s_ and ϕ_B,s_ in the shell region and 0 and ϕ_B,c_ in the core region, respectively. The result is given by

(B13) $$\frac{a^{2}}{k_{B}T}\gamma =\sqrt{\frac{\phi_{A,s}+\phi_{B,s}+\phi_{B,c}}{3}\left\{f\left[\frac{1}{2}\phi_{A,s},\frac{1}{2}\left(\phi_{B,s}+\phi_{B,c}\right)\right]-\frac{1}{2}\left[f\left(\phi_{A,s},\phi_{B,s}\right)+f\left(0,\phi_{B,c}\right)\right]\right\}}$$

where the function *f*(*x*, *y*) is defined by

(B14) $$f\left(x,y\right)\equiv \left[\ln\left(1-x-y\right)+\chi_{\mathrm{AS}}x+\chi_{\mathrm{BS}}y\right]\left(1-x-y\right)+\chi_{\mathrm{AB}}xy$$

There is one more interface between the shell and solvent regions in Figure 1, but this interface is not so sharp that we did not consider its interfacial Gibbs energy.

Flory [29] extended the Flory–Huggins theory [16] to solutions of a semiflxible polymer with energetically unfavorable “bend” conformations and demonstrated that the equilibrium degree of the bending of the chain is independent of the polymer concentration. This result indicates that the chain stiffness does not contribute to the mixing Gibbs energy Δ*G* because of the cancelation of the bending energy in the solution and bulk states. Therefore, the above formulations of Δ*S* and Δ*H* can be applied to semiflexible polymer solutions.
